# Lectures in the Medical Department of Transylvania University

**Published:** 1828-07

**Authors:** C. W. Short

**Affiliations:** Dean. Lexington, Kentucky


18. *Transylvania University—Medical Department.*—The Medical Lectures will commence, as usual, on the first Monday of November, and terminate on the first Saturday of March.

BENJAMIN W. DUDLEY, M. D., on Anatomy and Surgery.
CHARLES CALDWELL, M. D., Institutes and Clinical Practice.
JOHN E. COOKE, M. D., Theory and Practice of Medicine.
WILLIAM H. RICHARDSON, M. D., Obstetricks and Diseases of Women and Children.
CHARLES W. SHORT, M. D., Materia Medica and Medical Botany.
JAMES BLYTHE, D. D., Chemistry and Pharmacy.

Each Professor lectures daily, Sabbaths excepted. The Ticket to the anatomical and surgical course is $20; each of the others is $15. Matriculation, with the use of the Library, $5. The Graduation Fee is $20.

C. W. SHORT, M. D., DEAN.

*Lexington, Kentucky, July* 28th, 1828.

